# Disrupted Brain Network Efficiency and Decreased Functional Connectivity in Multi-sensory Modality Regions in Male Patients With Alcohol Use Disorder

**DOI:** 10.3389/fnhum.2018.00513

**Published:** 2018-12-21

**Authors:** Yaqi Wang, Yilin Zhao, Hongyan Nie, Changsheng Liu, Jun Chen

**Affiliations:** ^1^Department of Radiology, Renmin Hospital of Wuhan University, Wuhan, China; ^2^Department of Radiology, Tongji Hospital, Tongji Medical College, Huazhong University of Science and Technology, Wuhan, China

**Keywords:** alcohol use disorder, graph theory, small-world, efficiency, multi-sensory modalities

## Abstract

**Background**: Recent studies have reported altered efficiency in selective brain regions and functional networks in patients with alcohol use disorder (AUD). Inefficient processing can reflect or arise from the disorganization of information being conveyed from place to place. However, it remains unknown whether the efficiency and functional connectivity are altered in large-scale topological organization of patients with AUD.

**Methods**: Resting-state functional magnetic resonance imaging (rsfMRI) data were experimentally collected from 21 right-handed males with AUD and 21 right-handed, age-, gender- and education-matched healthy controls (HCs). Graph theory was used to investigate inter-group differences in the topological parameters (global and nodal) of networks and inter-regional functional connectivity. Correlations between group differences in network properties and clinical variables were also investigated in the AUD group.

**Results**: The brain networks of the AUD group showed decreased global efficiency when compared with the HC group. Besides, increased nodal efficiency was found in the left orbitofrontal cortex (OFC), while reduced nodal efficiency was observed in the right OFC, right fusiform gyrus (FFG), right superior temporal gyrus, right inferior occipital gyrus (IOG), and left insula. Moreover, hypo-connectivity was detected between the right dorsolateral prefrontal cortex (DLPFC) and right superior occipital gyrus (SOG) in the AUD group when compared with the HC group. The nodal efficiency of the left OFC was associated with cognitive performance in the AUD group.

**Conclusions**: AUD patients exhibited alterations in brain network efficiency and functional connectivity, particularly in regions linked to multi-sensory modalities. These disrupted topological properties may help to obtain a more comprehensive understanding of large-scale brain network activity. Furthermore, these data provide a potential neural mechanism of impaired cognition in individuals with AUD.

## Introduction

Alcohol use disorder (AUD) is defined as a progressive and potentially fatal chronic disease that is characterized by a loss of control of alcohol consumption and constant preoccupation with the ingestion of alcoholic beverages despite adverse consequences. Considerable effort has been made to gain a better understanding of AUD characteristics, particularly those associated with brain and cognitive impairments (Maurage et al., 2013b).

Many researchers have used structural magnetic resonance imaging (sMRI) and functional MRI (fMRI) to assess changes in different brain regions, inter-regional circuits, and functional networks in patients with AUD. A previous meta-analysis has demonstrated that patients with AUD exhibit significant reductions of gray matter (GM) in the corticostriatal-limbic circuits [including the bilateral insula, superior temporal gyrus, striatum, dorsal lateral prefrontal cortex (DLPFC), precentral gyrus, anterior cingulate cortex, left thalamus, and right hippocampus], which might indicate functional deficits in craving behavior (Yang et al., 2016). Furthermore, patients with AUD have altered spontaneous brain function and functional connectivity networks. For example, an independent component analysis (ICA) study has revealed higher functional connectivity within the fronto-parietal cognitive control network and motivational network [striatum and orbitofrontal cortex (OFC)] in patients with AUD, which implies that increased connectivity is required for the increasing demand of cognitive control (Jansen et al., 2015). Moreover, Weiland et al. (2014) have revealed that patients with AUD exhibit diminished connectivity in the left executive control network, basal ganglia, and primary visual networks, which can weaken the capacity to integrate information in executive control, sensorimotor, and visual regions.

The cognitive repercussions of alcohol dependence are well documented (Stavro et al., 2013). Cognitive (sensory) innovativeness is the preference for engaging in new experiences with the objective of stimulating the mind (senses; Venkatraman and Price, 1990). Numerous studies have explored multi-sensory processing deficits in patients with AUD, particularly in emotional, visual, and auditory processing (He et al., 2013; Brion et al., 2017). The same experiment imagery for seven different sensory modalities (visual, auditory, kinesthetic, olfactory, gustatory, tactile, and somatic), triggered by visually presented sentences, was investigated in an fMRI study (Olivetti Belardinelli et al., 2004). Olivetti Belardinelli et al. (2009) have revealed that the level of imagery vividness in different sensory modalities is related to differences of BOLD activity in modality-specific cortices. Activation of several brain regions in different sensory modalities has been identified in some research articles, such as occipital (visual), superior temporal gyrus (auditory), post-central gyrus (somatic), anterior insula (gustatory and olfactory), fusiform gyrus (FFG; visual and semantic), and OFC (olfactory and visual; Olivetti Belardinelli et al., 2009; Li et al., 2010; Campo et al., 2016; Harris et al., 2016; Kaufman et al., 2016; Zhang et al., 2018). Previous studies have indicated that patients with AUD demonstrate significant GM reductions in these multi-sensory modality regions (superior, middle frontal gyrus, occipital cortex, superior temporal gyrus, and insula; Senatorov et al., 2015; Yang et al., 2016). Furthermore, Vergara et al. (2017) have revealed hypo-connectivity in the precuneus, post-central gyrus, insula, and visual cortex, which suggests that there is a reduction in interoceptive awareness in patients with AUD.

Intact cognitive functioning draws on an efficient interaction of several brain regions, each specialized for information processing in certain domains (Bressler and Menon, 2010; Binder et al., 2017). The disruption of functional brain networks may be one reason for the inefficient processing in patients with AUD (Stanley et al., 2015). Graph theory analysis (GTA) offers a powerful tool to characterize and quantify global and local network topological parameters in large-scale brain network architecture. High clustering coefficients and short characteristic path lengths indicates that the small-world network is advantageous when compared with regular and random networks in segregated and distributed information processing (Bassett and Bullmore, 2006). In graph theory, both the structural and functional networks of the brain possess small-world networks (Bullmore and Sporns, 2009). Disrupted topological organization of small-world networks has been found in patients with AUD (Cao et al., 2014; Sjoerds et al., 2017). However, previous studies mainly focused on unit-modal regions, specific brain systems, or between different systems (Chanraud et al., 2011; Sjoerds et al., 2017). Hence, alterations in the efficiency and functional connectivity of large-scale topological organization in patients with AUD remains unknown.

In the present study, we applied GTA to resting-state fMRI (rsfMRI) to examine alterations in the topological organization of brain networks at the large-scale level (global, nodal, and connectivity) in patients with AUD. We hypothesized that these patients showed abnormal network efficiency and functional connectivity, particularly in multi-sensory modality regions [OFC, DLPFC, insula, FFG, superior temporal gyrus and inferior occipital gyrus (IOG)]. In addition, the disrupted network properties of large-scale brain networks in AUD patients were related to the changes in cognitive ability.

## Materials and Methods

### Participants

The protocol was approved by the Medical Ethical Committee of Renmin Hospital of Wuhan University and the studies were carried out in accordance with the Declaration of Helsinki. Subjects were fully informed about the measurement and MRI scanning procedures in the study. Written informed consent was given and obtained by all subjects.

Twenty-one right-handed males with AUD and 21 right-handed, age-, gender- and education-matched healthy controls (HCs) who did not use alcohol participated in this study. All participants were aged between 35 years and 60 years old and did not smoke. They underwent a screening process that included medical history, and physical and neurological examinations, assessed by a psychiatrist. Any physical illnesses, recorded in personal history or upon examination, were excluded. Furthermore, serious neurological or mental disorders that are associated with diseases other than AUD, organic brain disorders, serious physical illness, abuse, substance dependence other than alcohol, or any contraindications for MRI were included in exclusion criteria for all participants.

Patients with AUD were recruited from the primary-care outpatient department of Renmin Hospital of Wuhan University. Inclusion criteria were: (1) history of alcohol dependence history for 10–30 years, and daily alcohol consumption of 300–500 ml (standard, pure ethanol); (2) Diagnostic and Statistical Manual of Mental Disorders: Fifth Edition (DSM-5) criteria, Michigan Alcoholism Screening Test (MAST) score ≥6, and Alcohol drinking scale (ADS) score ≥14, indicating moderate or severe alcohol dependence (Conley, 2006); (3) abstinence for at least 3 weeks before participating in the study; and (4) no prior history of treatment with AUD and other psychoactive medication. HCs were defined as individuals who never drink or rarely drink (such as holiday drinking, <1 standard unit per time). All HCs should met the following criteria: (1) one standard drink unit, defined as 14 g of pure ethanol (Pilatti et al., 2017); and (2) MAST <6, and ADS scores <14, indicating mild or no alcohol dependence (Conley, 2006).

### Cognitive and Alcohol Level Evaluation

Cognitive function was evaluated using the Mini-Mental State Examination (MMSE). Levels of alcohol-related problems were assessed using the MAST and ADS in AUD group.

### Data Acquisition and Preprocessing

All participants were scanned in a 3.0T GE Silent MR scanner (Discovery 750w Silent MR, GE Healthcare, Milwaukee, WI, USA) in Renmin Hospital of Wuhan University, Wuhan, China. Participants were instructed to remain still, with their eyes closed without falling asleep. Conventional T1-weighted and T2-weighted images were collected to exclude macrostructural brain lesions that might affect brain function or microstructure.

A gradient-recalled echo-planar imaging (EPI) sequence was used in functional imaging. The acquisition parameters were as follows: echo time (TE) = 25 ms; repetition time (TR) = 2,000 ms; field of view (FOV) = 240 × 240 mm^2^, slice thickness = 4 mm, slice gap = 0.6 mm; matrix = 64 × 64; flip angle = 90°, volumes = 240; and 32 slices. T1-weighted 3D images were set for anatomical reference, and structural T1-weighted images were acquired in the sagittal orientation using the following parameters: TR = 7.5 ms, TE = 2.8 ms, FOV = 256 × 256 mm^2^, slice thickness = 1 mm, slices = 156, no slice gap, matrix = 256 × 256, flip angle = 12°, and voxel size = 1 × 1 × 1 mm^3^.

Data were preprocessed using the Network Construction module in Graph Theoretical Network Analysis (GRETNA) toolbox^1^ (Wang et al., 2015). The first 10 images were excluded to ensure steady states; the remaining 230 images were processed. Images were corrected for slice timing and realigned to the middle slice for head movement correction. Participants with head movement exceeding 2.0 mm translation or 2.0° rotation in any direction were excluded; however, no participant was excluded due to excessive head motion. Mean frame-wise displacement (FD) was computed (Patel and Bullmore, 2016). Next, functional images were normalized to the standard Montreal Neurological Institute (MNI) space and resampled to voxel size of 3 × 3 × 3 mm^3^. Images were not smoothed to avoid introducing artificial local spatial correlations (Liu et al., 2015a). In addition, linear trend removal and temporal bandpass filtering (0.01–0.08 Hz) were performed to reduce the effects of low-frequency drifts and high-frequency physiological noises. Finally, the nuisance signals of the 24-parameter head motion parameters, and white matter and cerebral spinal fluid signals were regressed out.

### Network Construction

In graph theory, the undirected network is regarded as a graph consisting of a series of nodes, and edges of connected nodes. The automated anatomical labeling (AAL) template (including 90 cerebral and 26 cerebellar regions) was used to divide the brain, and each region was defined as a node (Liu et al., 2016). In the current study, we focused on cerebral regions in patients with AUD, which equaled 90 nodes. Pearson’s correlation coefficients were calculated between the regional mean time series of all possible pairs of the 90 brain regions as edges in the network, resulting in a 90 × 90 Pearson’s correlation matrix for each participant, which was converted into binarized matrices. A Fisher’s r-to-z transformation was performed to translate the individual correlation maps into z-scored maps to improve normality (Liu et al., 2017).

### Network Analysis Threshold Selection

We set a range of sparsity thresholds to construct the undirected network. In this study, sparsity was defined as the number of edges on the graph divided by the maximum possible number of edges. The mean degree (the mean number of connections linked to a node) for each network was larger than 2log(*N*; Achard et al., 2006), where *N* is the number of nodes (here, *N* = 90); and the resultant network has sparse and distinguishable properties compared with the degree-matched random network (He et al., 2007; Wang et al., 2009). According to these rules, we selected the sparsity range of 10%–50% with an interval of 1%.

### Network Metrics

Global and local network attributes were analyzed per participant, and the attribute values were calculated using the Network Analysis module in GRETNA toolbox. Global network attributes included small-world properties and network efficiency, which involved the clustering coefficient (Cp), characteristic path length (Lp), normalized clustering coefficient (γ), normalized characteristic path length (*λ*), small-worldness (δ), local efficiency (Eloc), and global efficiency (Eg). A small-world network with a significantly higher Cp and similar Lp compared with random networks should meet following criteria: γ = Cp_real_/Cp_rand_ > 1, and *λ* = Lp_real_/Lp_rand_ ≈ 1, or δ = γ/*λ* > 1 (Chen et al., 2018). Nodal efficiency was regarded as a local network attribute in this study. To eliminate the effect of threshold selection, we further calculated the area under the curve (AUC) value for each network metric, which provided a summarized scalar for brain topological properties.

### Statistical Analysis

Two-sample *t*-tests were used to investigate the differences in the demographic, clinical, and AUC values of each metric between groups. Small-world properties (Cp, Lp, γ, *λ*, and δ), network efficiency (e.g., Eloc), nodal characteristics (nodal efficiency), and unconcerned confounding factors (age, years of education, and mean FD) for each network property were regressed out as covariates. In addition, two-sample *t*-tests were employed for each pair of regions to investigate the changes of functional connectivity in the AUD group. A false discovery rate (FDR) correction method was used at a *p*-value of 0.05 to correct for multiple comparisons. Linear correlational analyses were used to analyze the relationship between group differences in network properties and clinical variables with age, education level, and head motion as covariates. Differences were considered significant if *p* < 0.05.

## Results

### Demographic and Clinical Characteristics

Participant characteristics were described in Table 1. There were no significant differences in gender, age, education level, or handedness between the AUD and HC groups. However, the AUD group had significantly lower MMSE scores (*p* < 0.05) than the HC group.

**Table 1:** Demographics and clinical characteristics of patients in the HC group and AUD group.

Variables	HC group (*n* = 21)	AUD group (*n* = 21)	*p* value
Gender (M/F)	21/0	21/0	-
Age (years)	48.29 ± 7.73	44.86 ± 8.33	0.719
Education (years)	11.29 ± 2.49	13.43 ± 3.23	0.143
Handedness (R/L)	21/0	21/0	-
Duration of alcohol use (years)	NA	27.14	-
MAST	NA	9.91 ± 2.19	-
ADS	NA	17.43 ± 2.16	-
MMSE	28.00 ± 1.26	23.91 ± 2.59	0.003
Head motion (mm)	0.057 ± 0.047	0.044 ± 0.036	0.692

*Abbreviations: HCs, healthy controls; AUD, alcohol use disorder; MAST, Michigan Alcoholism Screening Test; ADS, Alcohol drinking scale; MMSE, Mini-Mental State Examination. The *p* value was obtained by two-sample *t*-test*.

### AUD-Related Alterations in Small-World Properties

**Figure 1:**
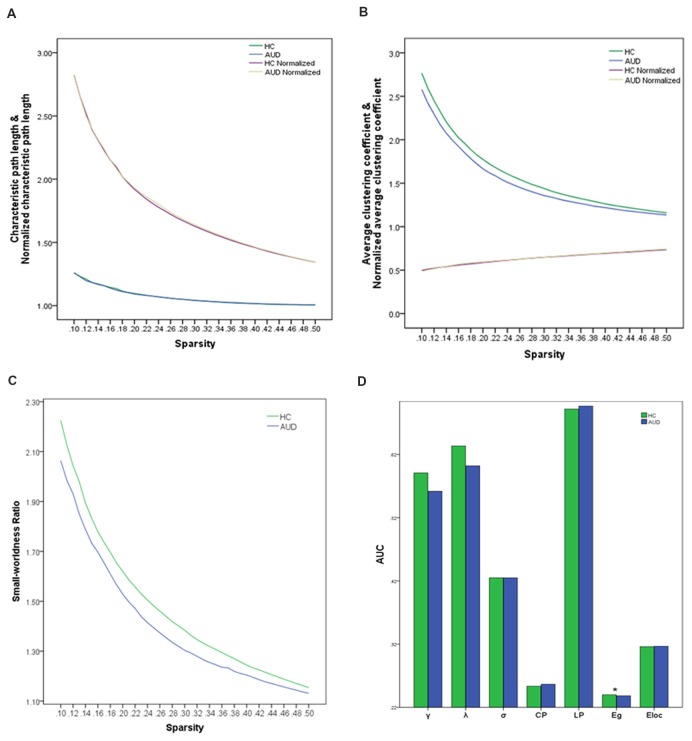
Global topological attributes of brain connectivity between the groups. **(A)** Characteristic and normalized characteristic path lengths. **(B)** Average and normalized average clustering coefficients. **(C)** Small-worldness. **(D)** AUC of the parameters of seven global topologies in two groups. Significant difference (asterisk) was only found in the Eg^AUC^ in the range of 10%–50% (HCs: 0.240 ± 0.005; AUD: 0.238 ± 0.008; *p* = 0.023). AUD, alcohol use disorder; HCs, healthy controls; AUC, the area under curve values; Eg^AUC^, the area under curve values of the global efficiency.

When the sparsity threshold was enhanced with an increasing step size of 1% in the sparsity, the other parameters, except normalized average clustering coefficients in the two groups, showed a monotonous decreasing trend. The normalized characteristic path lengths were close to 1 in both groups, indicating that the characteristic path length was similar to a random network (Figure 1A); however, the average clustering coefficient was substantially greater than the random network (Figure 1B). The small-world ratio was >1.1 over the entire range of sparsity thresholds, which further verified that the brain was a small-world network in both groups (Figure 1C). Two-sample *t*-tests indicated that the AUC of global efficiency (Eg^AUC^) showed significant differences between the groups (Figure 1D). The AUD group had a smaller Eg^AUC^ compared with the HC group (HC: 0.240 ± 0.005; AUD: 0.238 ± 0.008; *p* = 0.023). No significant (*p* > 0.05) group differences were identified for small-world parameters and local efficiency.

### AUD-Related Alterations in Nodal Efficiency

**Figure 2:**
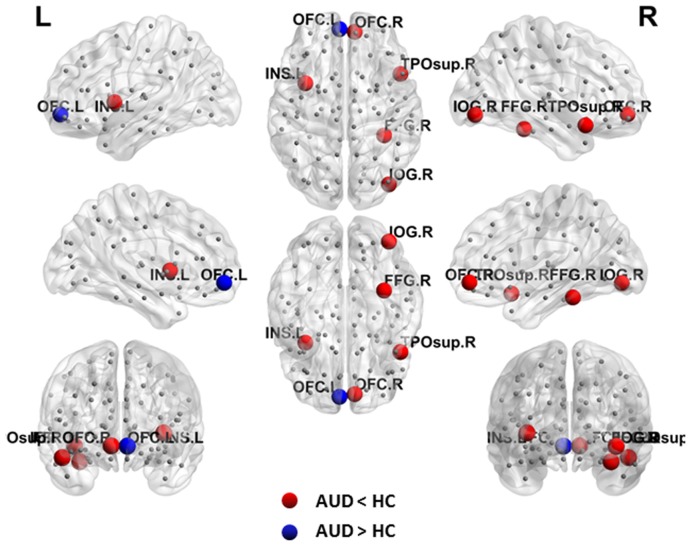
Abnormal nodal efficiency in brain functional networks in the AUD group. Blue dots indicate increased nodal efficiency and red dots indicate decreased nodal efficiency in the AUD group compared with the HC group. AUD, alcohol use disorder; HCs, healthy controls; L, left; R, right; OFC, orbitofrontal cortex; INS, insula; IOG, inferior occipital gyrus; FFG, fusiform gyrus; TPOsup, temporal pole: superior temporal gyrus.

Local topology was considered to be abnormal in the AUD group if two groups exhibited significant differences (*p* < 0.05, uncorrected) in nodal efficiency. The AUD group showed increased nodal efficiency in the left OFC, and decreased nodal efficiency in the right OFC, right FFG, right temporal pole: superior temporal gyrus, right IOG, and left insula when compared with the HC group (Table 2, Figure 2).

**Table 2:** Abnormal nodal efficiency in the AUD group as compared with the HC group.

	Brain region	*p* value
AUD > HC
	Left superior frontal gyrus, medial orbital	0.026
AUD < HC		
	Right superior frontal gyrus, medial orbital	0.030
	Right fusiform gyrus	0.018
	Right temporal pole: superior temporal gyrus	0.033
	Right inferior occipital gyrus	0.037
	Left insula	0.033

*AUD, alcohol use disorder; HCs, healthy controls*.

### AUD-Related Alterations in Functional Connectivity

**Figure 3:**
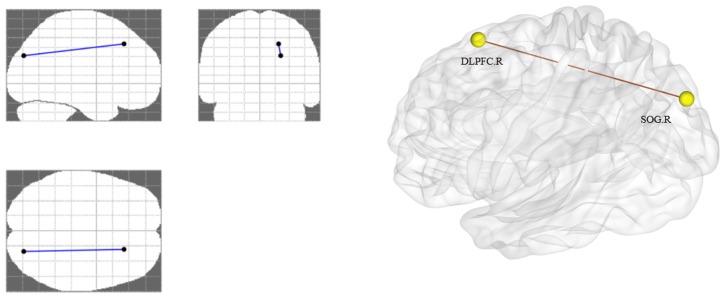
Altered functional connectivity in AUD group. The right DLPFC and right SOG showing a decreased functional connection in the AUD group compared with the HC group. AUD, alcohol use disorder; HCs, healthy controls; L, left; R, right; DLPFC, dorsolateral prefrontal cortex; SOG, superior occipital gyrus.

We identified a single connected network (*p* < 0.05, FDR corrected). One connection and two nodes were encompassed in the network (Table 3, Figure 3). In the network, the functional connectivity value exhibited a clear decline between the right DLPFC and right superior occipital gyrus (SOG) in the AUD group when compared with the HC group.

**Table 3:** Inter-regional changes and functional connectivity in the AUD group compared with the HC group.

Brain region A	Brain region B	*t* value
Right dorsolateral prefrontal cortex	Right superior occipital gyrus	3.58

*AUD, alcohol use disorder; HCs, healthy controls*.

### Relationships Between Network Measures and Clinical Variables

**Figure 4:**
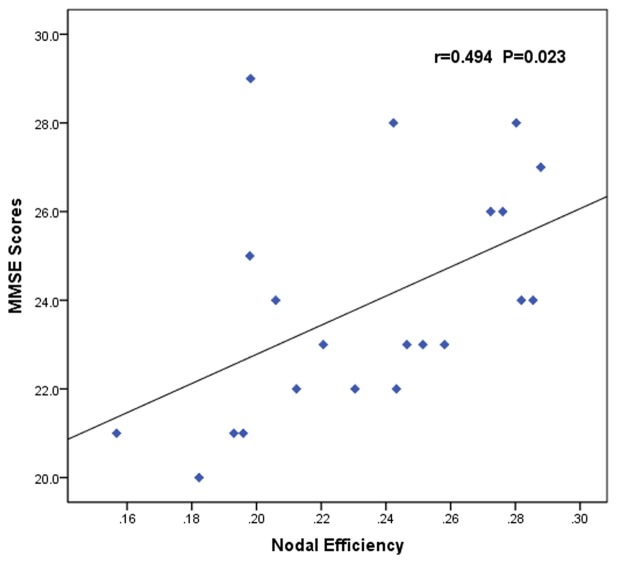
Correlation between abnormal brain functional networks and clinical variables of the AUD group. Scatter plots of the nodal efficiency of the left OFC against the MMSE scores (*r* = 0.494, *p* = 0.023). AUD, alcohol use disorder; OFC, orbitofrontal cortex; MMSE, Mini-Mental State Examination.

The nodal efficiency of left OFC was positively correlated with MMSE scores (*r* = 0.494, *p* = 0.023; Figure 4) in the AUD group. There were no significant correlations (*p* < 0.05) between the other network metrics of group differences and clinical variables.

## Discussion

This study used a multilevel approach to characterize the altered efficiency and functional connectivity in the multi-sensory modality regions in patients with AUD. Compared with the HC group, the abnormal network measurements of the AUD group within the range of 10%–50% were as follows: (1) abnormal global network measures parameters (smaller Eg^AUC^); (2) regions with altered node efficiency were mainly located in the motivational and face-voice integration networks; (3) decreased connection in the control and visual cortices; and (4) abnormal network efficiency was correlated with cognitive performance.

In line with these findings, small-world properties of functional networks were found in both groups, which reflected the economical properties (high efficiency information transmission at low cost; Cao et al., 2014; Sjoerds et al., 2017). However, the topology of the AUD group was altered when compared with the HC group. The relatively smaller Eg^AUC^ might indicate inefficient global integration and a tendency of shift toward random networks in the AUD group. In the AUD group, in addition to the decreased global efficiency, we found increased nodal efficiency in the left OFC, and decreased nodal efficiency in the right OFC, right FFG, right temporal pole: superior temporal gyrus, right IOG, and left insula.

The OFC is associated with emotive (Han et al., 2016), olfactory (Li et al., 2010), and visual (Chaumon et al., 2014; Kaufman et al., 2016) information processing and is an important hub of information transmission in the motivational network (Jansen et al., 2015). At the interface between sensory, memory, and affective processing, the OFC appears to be attuned to the associative content of visual information and plays a central role in visuoaffective prediction (Chaumon et al., 2014). Kaufman et al. (2016) have suggested that enhanced OFC activity at early stages during visual attention increases their top-down control over visual attention in healthy aging. In the AUD group, increased efficiency in the left OFC may be an attempt to compensate for alcohol-induced impairments. However, heavier damage may relate to extensive brain damage and weaken this compensatory activity. Drinking alters the balance of GABA in the OFC; GABA agonist treatment may alleviate OFC activation and desire for alcohol (Enoch, 2008).

The FFG is distributed in the face perception network (Freitas-Ferrari et al., 2010; Stevenson et al., 2011; Liu et al., 2015b) to process different types of visual information, such as the identification of faces and processing of words. Namely, the FFG shows a response to either words or faces. The right hemisphere mainly processes facial information (Pitcher et al., 2007), and word-selective regions are more distinct in the left hemisphere (Harris et al., 2016). These different effects are useful for separating drinkers and smokers (Vergara et al., 2017).

In addition, the region with altered node efficiency was distributed in the IOG, which is part of the visual cortex. One study has reported that the visual cortex activity is correlated with reported vividness, demonstrating that individual differences in mental imagery vividness are detectable *via* fMRI (Cui et al., 2007).

The superior temporal gyrus, which can process auditory information, is considered to be a critical area for audiovisual integration (Beaucousin et al., 2007; Campo et al., 2016). The results presented here indicated that hypo-activation of the FFG and superior temporal gyrus in the AUD group might present a cross-modal integration impairment in the face-voice integration network. Impairment of the specific electrophysiological components and functional connectivity associated with audiovisual integration in patients with AUD confirmed this cross-modal deficit (Maurage et al., 2008, 2013a).

Sensory and affective processing are associated with insula activation (Uddin et al., 2014). There is a transformation from sensory to affective representations in auditory modality, and the insula is an important site for multimodal convergence (Zhang et al., 2018). The left insula also processes higher gustatory information by receiving signals from regions in the frontal cortex that may mediate top-down control from long-term memories (Olivetti Belardinelli et al., 2009). Hypoconnectivity within the insula has been previously reported and hypothesized to play a role in alcohol relapse due to diminished substance use awareness (Vergara et al., 2017).

Reduced network efficiency can be attributed to impaired communication between brain regions, and differences between groups in the edge properties that were assessed. The DLPFC showed reduced edge connectivity in patients with AUD in this study. This region is associated with top-down attentional focus (Weiland et al., 2014). In addition, the DLPFC is involved in cognitive control, such as self-monitoring and processing auditory information (Salehinejad et al., 2017). Bagga et al. (2014) have indicated that impairments in visual processing abilities in patients with AUD are related to fronto-occipital white matter microstructural alterations. In present the study, patients portrayed a diminished connectivity between the DLPFC and SOG, which would weaken the top-down regulatory mechanism and may be related to the diminished auditory and visual processing abilities.

Several limitations of the current study should be considered. First, according to Cloninger’s typology, alcohol dependence was classified into early-onset (age of onset <25 years) and late-onset (age of onset >25 years) subgroups (Kist et al., 2014; Ghosh et al., 2016). Each type of alcohol dependence has different neuromechanical presentations, time courses, structures and functions of the brain, and neurocognitive function (Kist et al., 2014; Holla et al., 2017). For example, the current study reports different results from Holla et al. (2017), which have found reduced clustering, small-worldness, and local efficiency, and preserved path lengths and global efficiency in early-onset patients with AUD. Nonetheless, right lateralized deficits are found in both studies. However, comparing different ages can lead to inaccuracies, and it is unclear how age mediates graph-theoretical measures. Future research should classify patients with AUD into subgroups, and longitudinal investigations are necessary. Second, the Severity of Alcohol Dependence Questionnaire (SAD-Q) and the AUD Identification Test (AUDIT) items were generated originally to operationalize a specific conceptual or theoretical framework. However, the MAST captures a large single factor of general alcoholic impairment in a more effective way (Conley, 2006). To the best of our knowledge, there are no studies addressing the relationship between brain function and screening instruments for alcohol dependence. However, the absence of results obtained from a second screening instrument, such as the AUDIT, would not provide a more precise evaluation of individuals with AUD. Third, group differences in global, nodal efficiency, and the association between nodal efficiency and MMSE scores varied between groups when considered at an uncorrected threshold. Patients were recruited from the primary-care outpatients department, from those who had not primarily sought help for alcohol-related problems. This might lead to weaker contrasts between the AUD and HC groups. Therefore, future research should classify patients based on their related clinical symptoms. In addition, the sample size in this study is small, therefore future should use larger sample sizes to increase the statistical power. Finally, the neural basis of mental image generation has been investigated *via* fMRI in the sensory modalities, showing the involvement in modality-specific and non-specific areas (Olivetti Belardinelli et al., 2004, 2009). However, rsfMRI is stationary, which will limit its ecological validity for because real-life situations are based on dynamic multi-sensory expressions.

## Conclusion

In this rsfMRI study, GTA was applied to investigate the topological properties in patients with AUD. The findings suggested the involvement of abnormal network efficiency and decreased functional connectivity in the multi-sensory modality regions in patients with AUD. The motivational (OFC) and face-voice integration (FFG and superior temporal gyrus) networks, control system (DLPFC), and visual cortex (inferior and SOG) were associated with alcohol-related abnormalities. Thus, these disrupted topological properties can help obtain a more comprehensive understanding of large-scale brain network activity in patients with AUD. Furthermore, they might provide a potential neural mechanism of impaired cognition in AUD individuals.

## Author Contributions

JC and YZ contributed to the study conception and design, revised the manuscript. YW, HN, and CL collected the original imaging data. YW, HN, and YZ analyzed and interpreted experimental results. YW and YZ drafted the manuscript. All authors have approved the final version of the manuscript.

## Conflict of Interest Statement

The authors declare that the research was conducted in the absence of any commercial or financial relationships that could be construed as a potential conflict of interest.

## Footnotes

^1^https://www.nitrc.org/projects/gretna
